# Supplementary figures and images for: The five homologous CiaR-controlled Ccn sRNAs of Streptococcus pneumoniae modulate Zn-resistance
Source: PLoS Pathog. 2024 Oct 3;20(10):e1012165. doi: 10.1371/journal.ppat.1012165 (PMC11478796; doi:10.1371/journal.ppat.1012165)

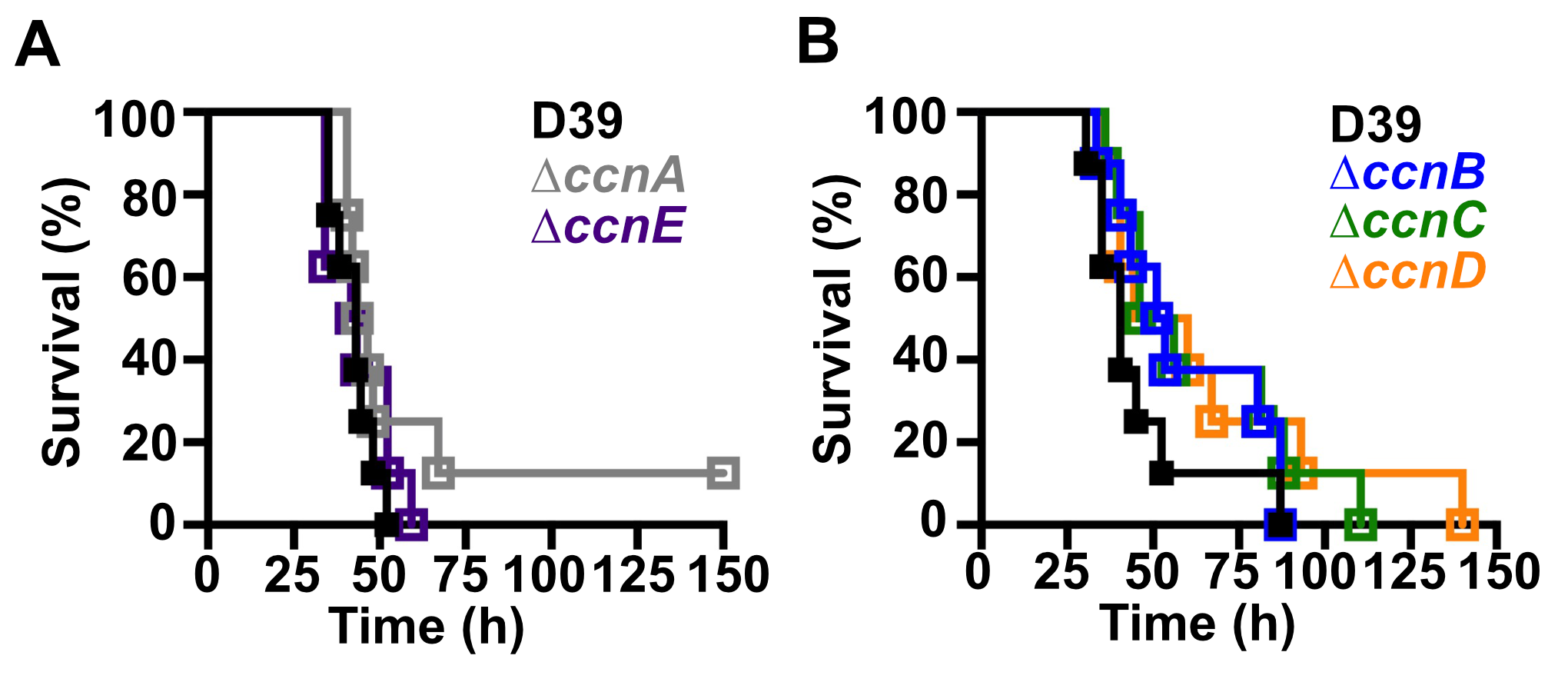

Supplement: S1 Fig — Survival curve of ICR outbred mice after infection with ~107 CFU in a 50 μL inoculum of the following S. pneumoniae strains: (A) IU781 (D39), NRD10073 (ΔccnA), and NRD10077 (ΔccnE); (B) IU781 (D39), NRD10074 (ΔccnB), NRD10075 (ΔccnC), and NRD10076 (ΔccnD). Eight mice were infected per strain. Disease progression of animals was monitored, the time at which animals reached a moribund state was recorded, and these mice were subsequently euthanized as described in Materials and Methods. A survival curve was generated from this data and analyzed by Kaplan-Meier statistics and log rank test to determine P-values. (TIF) [file ppat.1012165.s001.tif]

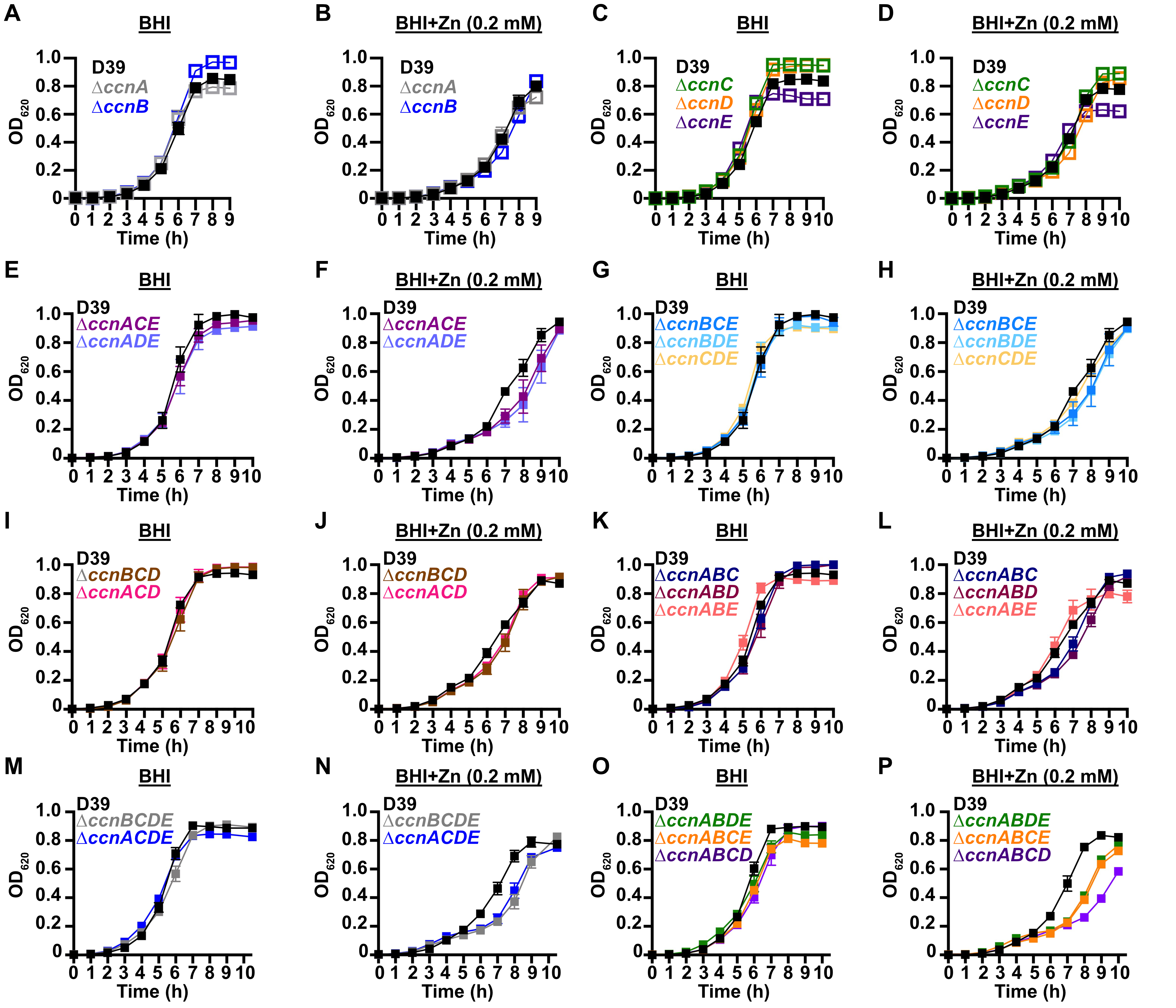

Supplement: S2 Fig — Growth characteristics at 37°C under an atmosphere of 5% CO2 in BHI broth alone (A,C, E, G, I, K, M, O) or with 0.2 mM ZnSO4 (B, D, F, H, J, L, N, P) of the following strains: (A, B) IU781 (D39), NRD10073 (ΔccnA), and NRD10074 (ΔccnB); (C, D) IU781 (D39), NRD10075 (ΔccnC), NRD10076 (ΔccnD), and NRD10077 (ΔccnE); (E, F) IU781 (D39), NRD10165 (ΔccnACE), and NRD10166 (ΔccnADE); (G, H) IU781 (D39), NRD10376 (ΔccnBCE), NRD10379 (ΔccnBDE), and NRD10380 (ΔccnCDE); (I, J) IU781 (D39), NRD10081 (ΔccnBCD), and NRD10084 (ΔccnACD); (K, L) IU781 (D39), NRD10372 (ΔccnABC), NRD10373 (ΔccnABD), and NRD10374 (ΔccnABE); (M, N) IU781 (D39), NRD10085 (ΔccnBCDE), and NRD10174 (ΔccnACDE); (O,P) IU781 (D39), NRD10172 (ΔccnABCE), NRD10173 (ΔccnABDE), and NRD10175 (ΔccnABCD). Each point on the graph represents the mean OD620 value from three independent cultures. Error bars, which in some cases are too small to observe in the graph, represent the standard deviation (SD). (TIF) [file ppat.1012165.s002.tif]

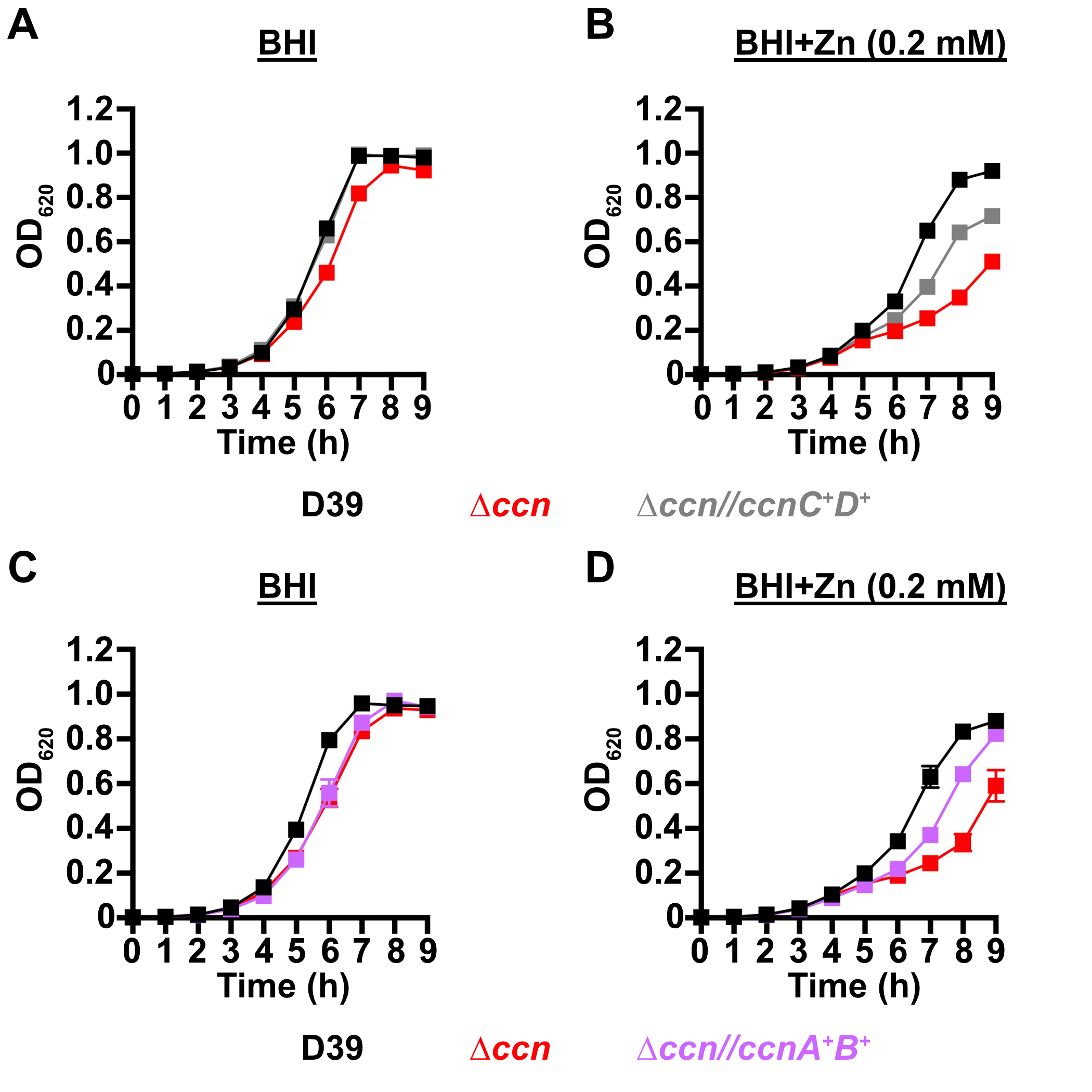

Supplement: S3 Fig — Growth characteristics at 37°C under an atmosphere of 5% CO2 in BHI broth alone (A, C) or with 0.2 mM ZnSO4 (B, D) of IU1781 (D39), NRD10176 (Δccn), and NRD10397 (Δccn//ccnC+D+) (A,B) or NRD10393 (Δccn//ccnA+B+) (C, D). Each point on the graph represents the mean OD620 value from three independent cultures. Error bars, which in some cases are too small to observe in the graph, represent the standard deviation (SD). (TIF) [file ppat.1012165.s003.tif]

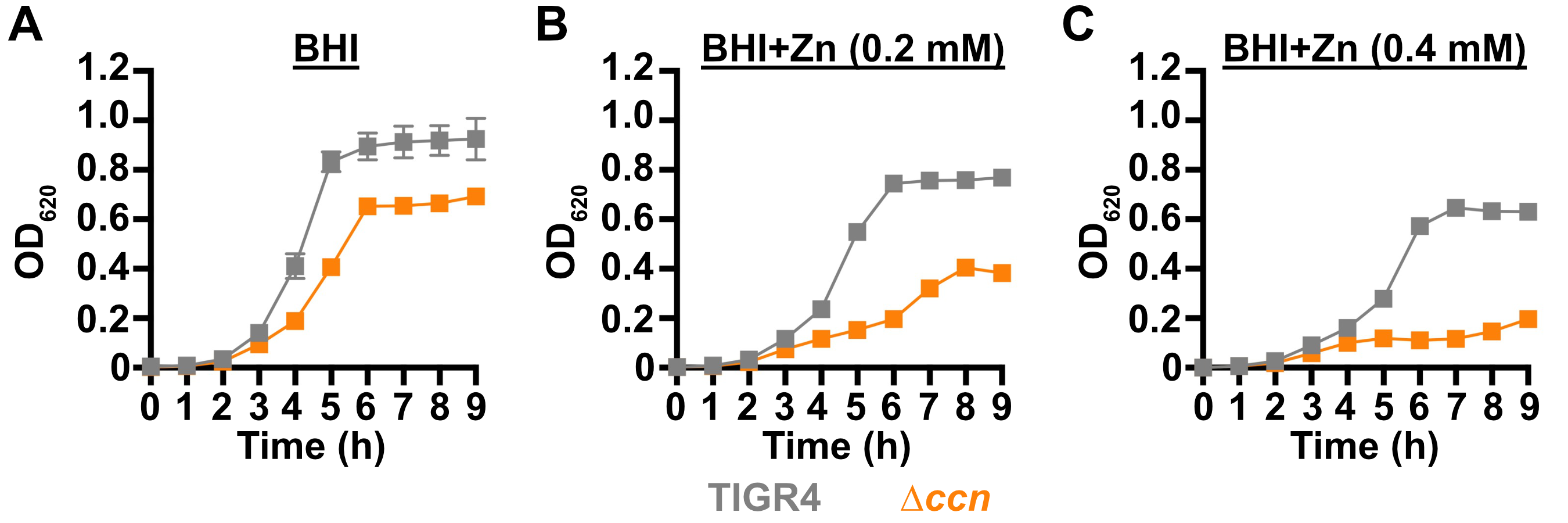

Supplement: S4 Fig — Growth characteristics at 37°C under an atmosphere of 5% CO2 in BHI broth alone (A) or with 0.2 mM (B) or 0.4 mM (C) ZnSO4 of NRD10311 (TIGR4; TIGR4 rpsL+-rpsG+-cat) and NRD10346 (Δccn; TIGR4 rpsL+-rpsG+-cat ΔccnABCDE). Each point on the graph represents the mean OD620 value from three independent cultures. Error bars, which in some cases are too small to observe in the graph, represent the standard deviation (SD). (TIF) [file ppat.1012165.s004.tif]

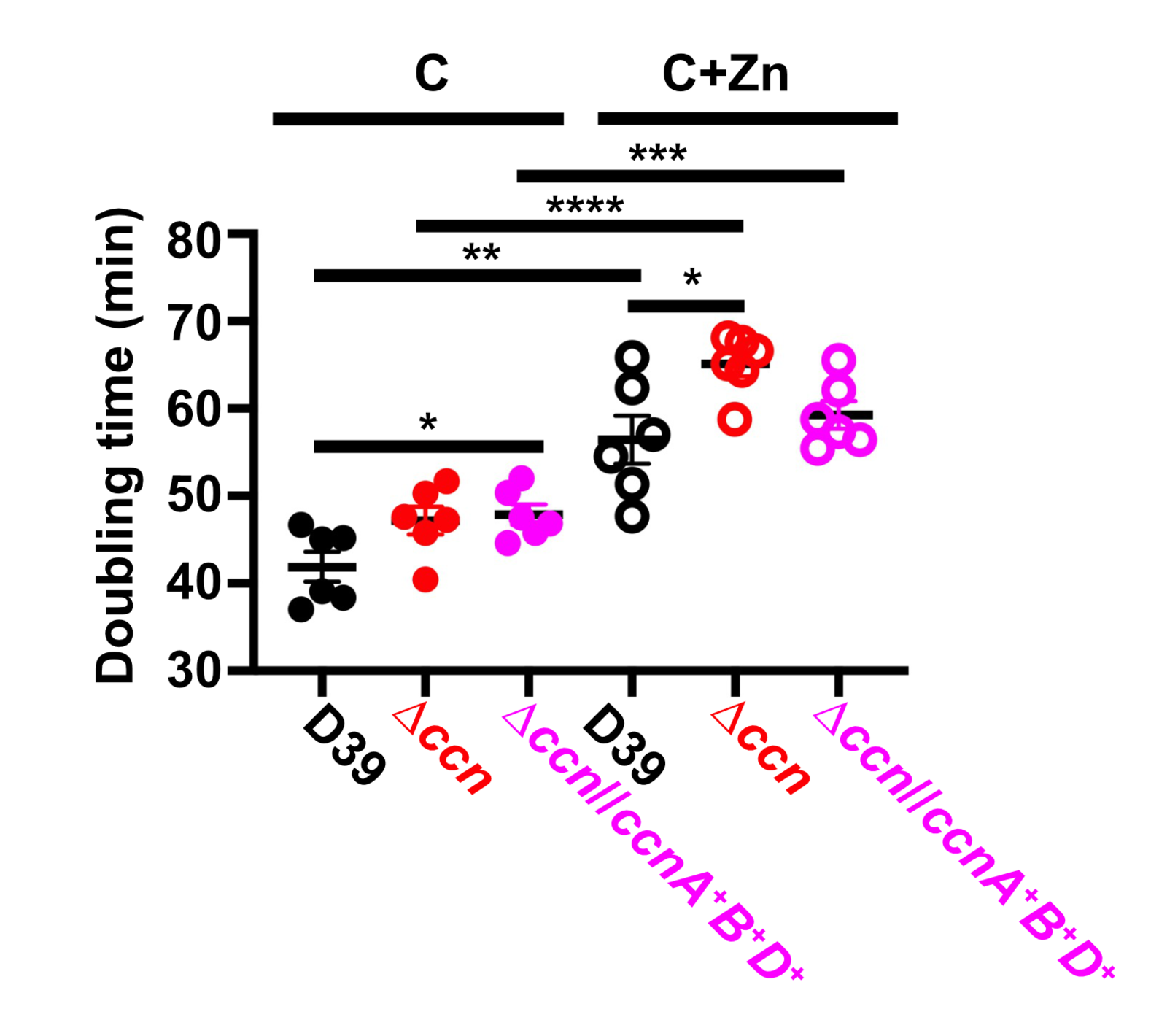

Supplement: S5 Fig — Shown are the mean doubling times during exponential growth of IU1781 (D39), NRD10176 (Δccn), and NRD10396 (Δccn//ccnA+B+D+) grown in C medium alone or supplemented with 0.2 mM ZnS04 as described in Materials and Methods. Doubling times for individual replicates are shown with solid lines indicating the mean of six different biological replicates and error bars denoting standard error of the mean (SEM). Statistical significance as determined by a Mann-Whitney test is indicate as * (P < 0.05), ** (P < 0.005), *** (P < 0.0005), or **** (P < 0.00005). (TIF) [file ppat.1012165.s005.tif]

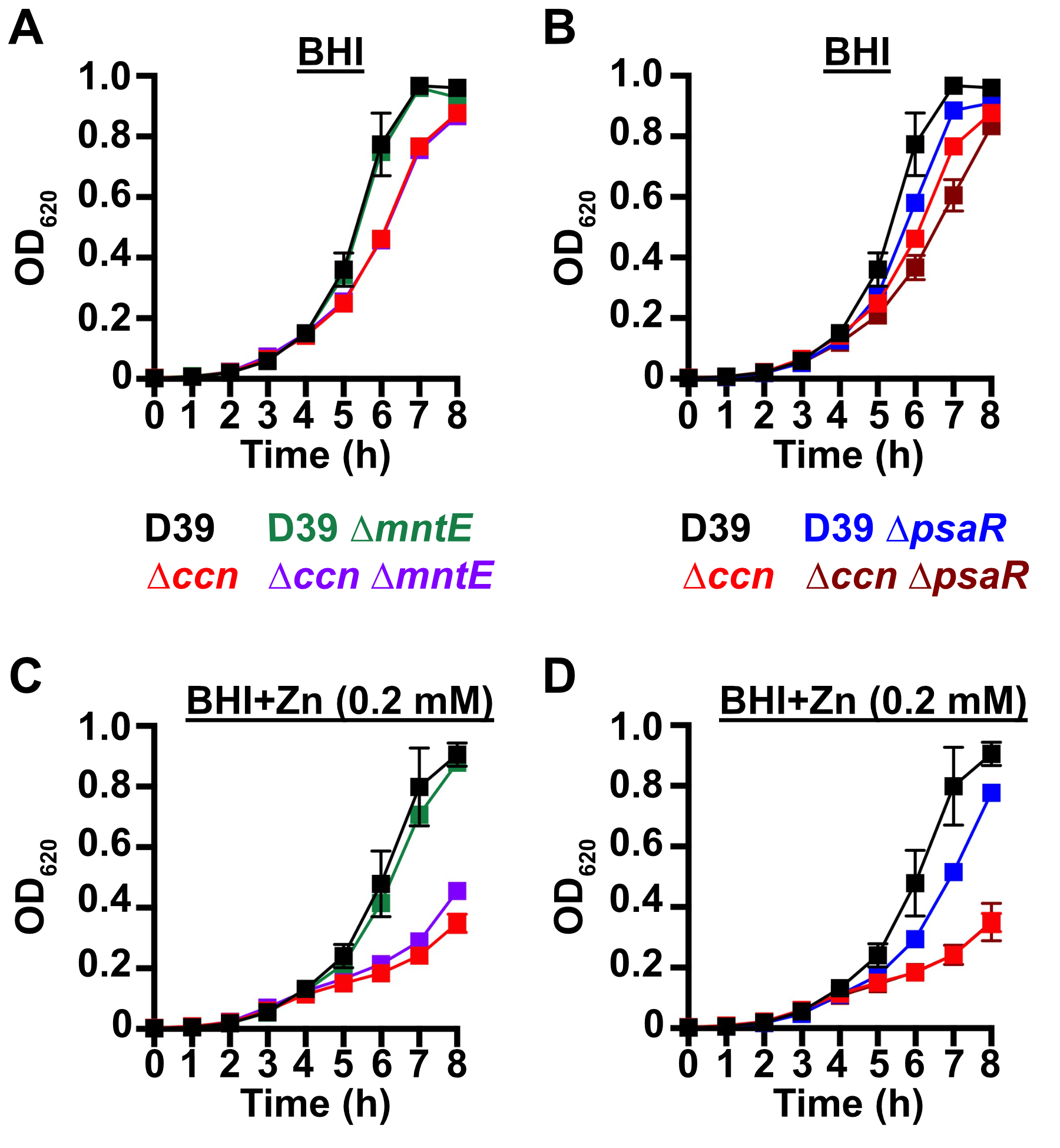

Supplement: S6 Fig — Growth characteristics at 37°C under an atmosphere of 5% CO2 in BHI broth alone (A, B) or with 0.2 mM ZnSO4 (C, D) of the following strains: (A, C) IU781 (D39), NRD10176 (ΔccnABCDE), NRD10448 (ΔmntE), and NRD10450 (ΔccnABCDE ΔmntE); (B, D) IU781 (D39), NRD10176 (ΔccnABCDE), NRD10447 (ΔpsaR), and NRD10450 (ΔccnABCDE ΔpsaR). Each point on the graph represents the mean OD620 value from three independent cultures. Error bars, which in some cases are too small to observe in the graph, represent the standard deviation (SD). (TIF) [file ppat.1012165.s006.tif]
