# Supplementary material for: The five homologous CiaR-controlled Ccn sRNAs of Streptococcus pneumoniae modulate Zn-resistance
Source: PLoS Pathog. 2024 Oct 3;20(10):e1012165. doi: 10.1371/journal.ppat.1012165 (PMC11478796; doi:10.1371/journal.ppat.1012165)
Supplement: S1 Table — (DOCX) [file ppat.1012165.s007.docx]

**S1 Table.** *S. pneumoniae* strains used in this study

| **Strain** | **Genotype (description)** | **Antibiotic resistance^b^** | **Reference or source** |
| --- | --- | --- | --- |
| K272 | D39 Δ*cps* Δ*pnp*::P_c_-[*kan rpsL^+^*] | Kan^R^ | [*1*] |
| IU1781 | D39 *rpsL1* | Str^R^ | [*2*] |
| IU5122 | D39 Δ*cps* *rpsL1* CEP::P_c_-[*kan rpsL^+^*] | Kan^R^ | [*3*] |
| IU5382 | D39 Δ*cps* CEP::P_fcsK_-*ftsE* | None | [*3*] |
| IU9681 | D39 Δ*cps* *hlpA hlpA-mkate-cat* | Cm^R^ | Malcolm Winkler |
| IU10508 | D39 Δ*cps* Δ*bgaA*::*kan-T_1_-T_2_-P_spd_1874_-lacZ* | Kan^R^ | [*4*] |
| IU11966 | TIGR4 | None | [*5, 6*] |
| IU12001 | TIGR4 Δ*cps* | None | [*7*] |
| NRD10068 | D39 *rpsL1* Δ*ccnA*::P_c_-[*kan rpsL^+^*] | Kan^R^ | This study |
| NRD10069 | D39 *rpsL1* Δ*ccnB*::P_c_-[*kan rpsL^+^*] | Kan^R^ | This study |
| NRD10070 | D39 *rpsL1* Δ*ccnC*::P_c_-[*kan rpsL^+^*] | Kan^R^ | This study |
| NRD10071 | D39 *rpsL1* Δ*ccnD*::P_c_-[*kan rpsL^+^*] | Kan^R^ | This study |
| NRD10072 | D39 *rpsL1* Δ*ccnE*::P_c_-[*kan rpsL^+^*] | Kan^R^ | This study |
| NRD10073 | D39 *rpsL1* Δ*ccnA* | Str^R^ | This study |
| NRD10074 | D39 *rpsL1* Δ*ccnB* | Str^R^ | This study |
| NRD10075 | D39 *rpsL1* Δ*ccnC* | Str^R^ | This study |
| NRD10076 | D39 *rpsL1* Δ*ccnD* | Str^R^ | This study |
| NRD10077 | D39 *rpsL1* Δ*ccnE* | Str^R^ | This study |
| NRD10078 | D39 *rpsL1* Δ*ccnB* Δ*ccnC*::P_c_-[*kan rpsL^+^*] | Kan^R^ | This study |
| NRD10079 | D39 *rpsL1* Δ*ccnB* Δ*ccnC* | Str^R^ | This study |
| NRD10080 | D39 *rpsL1* Δ*ccnB* Δ*ccnC* Δ*ccnD*::P_c_-[*kan rpsL^+^*] | Kan^R^ | This study |
| NRD10081 | D39 *rpsL1* Δ*ccnB* Δ*ccnC* Δ*ccnD* | Str^R^ | This study |
| NRD10082 | D39 *rpsL1* Δ*ccnA*::P_c_-[*kan rpsL^+^*] Δ*ccnC* Δ*ccnD* | Kan^R^ | This study |
| NRD10083 | D39 *rpsL1* Δ*ccnB* Δ*ccnC* Δ*ccnD* Δ*ccnE*::P_c_-[*kan rpsL^+^*] | Kan^R^ | This study |
| NRD10084 | D39 *rpsL1* Δ*ccnA* Δ*ccnC* Δ*ccnD* | Kan^R^ | This study |
| NRD10085 | D39 *rpsL1* Δ*ccnB* Δ*ccnC* Δ*ccnD* Δ*ccnE* | Str^R^ | This study |
| NRD10161 | D39 *rpsL1* Δ*ccnA* Δ*ccnE*::P_c_-[*kan rpsL^+^*] | Kan^R^ | This study |
| NRD10162 | D39 *rpsL1* Δ*ccnA* Δ*ccnE* | Str^R^ | This study |
| NRD10163 | D39 *rpsL1* Δ*ccnA* Δ*ccnC*::P_c_-[*kan rpsL^+^*] Δ*ccnE* | Kan^R^ | This study |
| NRD10164 | D39 *rpsL1* Δ*ccnA* Δ*ccnD*::P_c_-[*kan rpsL^+^*] Δ*ccnE* | Kan^R^ | This study |
| NRD10165 | D39 *rpsL1* Δ*ccnA* Δ*ccnC* Δ*ccnE* | Str^R^ | This study |
| NRD10166 | D39 *rpsL1* Δ*ccnA* Δ*ccnD* Δ*ccnE* | Str^R^ | This study |
| NRD10167 | D39 *rpsL1* Δ*ccnAB*::P_c_-[*kan rpsL^+^*] Δ*ccnC* Δ*ccnE* | Kan^R^ | This study |
| NRD10168 | D39 *rpsL1* Δ*ccnAB*::P_c_-[*kan rpsL^+^*] Δ*ccnD* Δ*ccnE* | Kan^R^ | This study |
| NRD10169 | D39 *rpsL1* Δ*ccnA* Δ*ccnD* Δ*ccnE* Δ*ccnC*::P_c_-[*kan rpsL^+^*] | Kan^R^ | This study |
| NRD10170 | D39 *rpsL1* Δ*ccnAB*::P_c_-[*kan rpsL^+^*] Δ*ccnC* Δ*ccnD* | Kan^R^ | This study |
| NRD10171 | D39 *rpsL1* Δ*ccnAB*::P_c_-[*kan rpsL^+^*] Δ*ccnC* Δ*ccnD* Δ*ccnE* | Kan^R^ | This study |
| NRD10172 | D39 *rpsL1* Δ*ccnAB* Δ*ccnC* Δ*ccnE* | Str^R^ | This study |
| NRD10173 | D39 *rpsL1* Δ*ccnAB* Δ*ccnD* Δ*ccnE* | Str^R^ | This study |
| NRD10174 | D39 *rpsL1* Δ*ccnA* Δ*ccnD* Δ*ccnE* Δ*ccnC* | Str^R^ | This study |
| NRD10175 | D39 *rpsL1* Δ*ccnAB* Δ*ccnC* Δ*ccnD* | Str^R^ | This study |
| NRD10176 | D39 *rpsL1* Δ*ccnAB* Δ*ccnC* Δ*ccnD* Δ*ccnE* | Str^R^ | This study |
| NRD10220 | TIGR4 *rpsL1* | Str^R^ | This study |
| NRD10225 | TIGR4 *rpsL1* Δ*ccnA*::P_c_-[*kan rpsL^+^*] | Kan^R^ | This study |
| NRD10230 | TIGR4 *rpsL1* Δ*ccnA* | Str^R^ | This study |
| NRD10247 | TIGR4 *rpsL1* Δ*ccnA* Δ*ccnB*::P_c_-[*kan rpsL^+^*] | Kan^R^ | This study |
| NRD10249 | TIGR4 *rpsL1* Δ*ccnA* Δ*ccnB* | Str^R^ | This study |
| NRD10251 | TIGR4 *rpsL1* Δ*ccnA* Δ*ccnB* Δ*ccnC*::P_c_-[*kan rpsL^+^*] | Kan^R^ | This study |
| NRD10254 | TIGR4 *rpsL1* Δ*ccnA* Δ*ccnB* Δ*ccnC* | Str^R^ | This study |
| NRD10257 | TIGR4 *rpsL1* Δ*ccnA* Δ*ccnB* Δ*ccnC* Δ*ccnD*::P_c_-[*kan rpsL^+^*] | Kan^R^ | This study |
| NRD10261 | TIGR4 *rpsL1* Δ*ccnA* Δ*ccnB* Δ*ccnC* Δ*ccnD* | Str^R^ | This study |
| NRD10265 | TIGR4 *rpsL1* Δ*ccnA* Δ*ccnB* Δ*ccnC* Δ*ccnD* Δ*ccnE*::P_c_-[*kan rpsL^+^*] | Kan^R^ | This study |
| NRD10266 | TIGR4 *rpsL1* Δ*ccnA* Δ*ccnB* Δ*ccnC* Δ*ccnD* Δ*ccnE* | Str^R^ | This study |
| NRD10306 | TIGR4 *rpsLK56T* Δ*ccnA*::P_c_-[*kan rpsL^+^*] | Kan^R^ | This study |
| NRD10311 | TIGR4 *rpsL^+^-rpsG^+^-cat* | Cm^R^ | This study |
| NRD10312 | TIGR4 *rpsLK56T* Δ*ccnA* | Str^R^ | This study |
| NRD10322 | TIGR4 *rpsLK56T* Δ*ccnA* Δ*ccnB*::P_c_-[*kan rpsL^+^*] | Kan^R^ | This study |
| NRD10324 | TIGR4 *rpsLK56T* Δ*ccnA* Δ*ccnB* | Str^R^ | This study |
| NRD10330 | TIGR4 *rpsLK56T* Δ*ccnA* Δ*ccnB* Δ*ccnC*::P_c_-[*kan rpsL^+^*] | Kan^R^ | This study |
| NRD10332 | TIGR4 *rpsLK56T* Δ*ccnA* Δ*ccnB* Δ*ccnC* | Str^R^ | This study |
| NRD10336 | TIGR4 *rpsLK56T* Δ*ccnA* Δ*ccnB* Δ*ccnC* Δ*ccnD*::P_c_-[*kan rpsL^+^*] | Kan^R^ | This study |
| NRD10340 | TIGR4 *rpsLK56T* Δ*ccnA* Δ*ccnB* Δ*ccnC* Δ*ccnD* | Str^R^ | This study |
| NRD10344 | TIGR4 *rpsLK56T* Δ*ccnA* Δ*ccnB* Δ*ccnC* Δ*ccnD* Δ*ccnE*::P_c_-[*kan rpsL^+^*] | Kan^R^ | This study |
| NRD10345 | TIGR4 *rpsLK56T* Δ*ccnA* Δ*ccnB* Δ*ccnC* Δ*ccnD* Δ*ccnE* | Str^R^ | This study |
| NRD10346 | TIGR4 *rpsL^+^-rpsG^+^-cat* Δ*ccnA* Δ*ccnB* Δ*ccnC* Δ*ccnD* Δ*ccnE* | Cm^R^ | This study |
| NRD10367 | D39 *rpsL1* Δ*ccnC* Δ*ccnAB*::P_c_-[*kan rpsL^+^*] | Kan^R^ | This study |
| NRD10368 | D39 *rpsL1* Δ*ccnD* Δ*ccnAB*::P_c_-[*kan rpsL^+^*] | Kan^R^ | This study |
| NRD10369 | D39 *rpsL1* Δ*ccnE* Δ*ccnAB*::P_c_-[*kan rpsL^+^*] | Kan^R^ | This study |
| NRD10370 | D39 *rpsL1* Δ*ccnE* Δ*ccnD*::P_c_-[*kan rpsL^+^*] | Kan^R^ | This study |
| NRD10371 | D39 *rpsL1* Δ*ccnB* Δ*ccnC* Δ*ccnE*::P_c_-[*kan rpsL^+^*] | Kan^R^ | This study |
| NRD10372 | D39 *rpsL1* Δ*ccnC* Δ*ccnAB* | Str^R^ | This study |
| NRD10373 | D39 *rpsL1* Δ*ccnD* Δ*ccnAB* | Str^R^ | This study |
| NRD10374 | D39 *rpsL1* Δ*ccnE* Δ*ccnAB* | Str^R^ | This study |
| NRD10375 | D39 *rpsL1* Δ*ccnE* Δ*ccnD* | Str^R^ | This study |
| NRD10376 | D39 *rpsL1* Δ*ccnB* Δ*ccnC* Δ*ccnE* | Str^R^ | This study |
| NRD10377 | D39 *rpsL1* Δ*ccnE* Δ*ccnD* Δ*ccnB*::P_c_-[*kan rpsL^+^*] | Kan^R^ | This study |
| NRD10388 | D39 *rpsL1* Δ*ccnE* Δ*ccnD* Δ*ccnC*::P_c_-[*kan rpsL^+^*] | Kan^R^ | This study |
| NRD10379 | D39 *rpsL1* Δ*ccnE* Δ*ccnD* Δ*ccnB* | Str^R^ | This study |
| NRD10380 | D39 *rpsL1* Δ*ccnE* Δ*ccnD* Δ*ccnC* | Str^R^ | This study |
| NRD10390 | D39 *rpsL1* CEP::P_c_-[*kan rpsL^+^*] | Kan^R^ | This study |
| NRD10391 | D39 *rpsL1* Δ*ccnAB* Δ*ccnC* Δ*ccnD* Δ*ccnE* CEP::P_c_-[*kan rpsL^+^*] | Kan^R^ | This study |
| NRD10393 | D39 *rpsL1* Δ*ccnAB* Δ*ccnC* Δ*ccnD* Δ*ccnE* CEP::T_1_-T_2_-*ccnA-ccnB* | Str^R^ | This study |
| NRD10394 | D39 *rpsL1* Δ*ccnAB* Δ*ccnC* Δ*ccnD* Δ*ccnE* CEP::T_1_-T_2_-*ccnC* | Str^R^ | This study |
| NRD10396 | D39 *rpsL1* Δ*ccnAB* Δ*ccnC* Δ*ccnD* Δ*ccnE* CEP::T_1_-T_2_-*ccnA-ccnB* Δ*bgaA*::*kan-T_1_-T_2_-ccnD* | Str^R^, Kan^R^ | This study |
| NRD10397 | D39 *rpsL1* Δ*ccnAB* Δ*ccnC* Δ*ccnD* Δ*ccnE* CEP::T_1_-T_2_-*ccnC* Δ*bgaA*::*kan-T_1_-T_2_-ccnD* | Str^R^, Kan^R^ | This study |
| NRD10441 | D39 *rpsL1* Δ*psaR*::P_c_-[*kan rpsL^+^*] | Kan^R^ | This study |
| NRD10442 | D39 *rpsL1* Δ*mntE*::P_c_-[*kan rpsL^+^*] | Kan^R^ | This study |
| NRD10443 | D39 *rpsL1* Δ*ccnAB* Δ*ccnC* Δ*ccnD* Δ*ccnE* Δ*psaR*::P_c_-[*kan rpsL^+^*] | Kan^R^ | This study |
| NRD10444 | D39 *rpsL1* Δ*ccnAB* Δ*ccnC* Δ*ccnD* Δ*ccnE* Δ*mntE*::P_c_-[*kan rpsL^+^*] | Kan^R^ | This study |
| NRD10447 | D39 *rpsL1* Δ*psaR* | Str^R^ | This study |
| NRD10448 | D39 *rpsL1* Δ*mntE* | Str^R^ | This study |
| NRD10449 | D39 *rpsL1* Δ*ccnAB* Δ*ccnC* Δ*ccnD* Δ*ccnE* Δ*psaR* | Str^R^ | This study |
| NRD10450 | D39 *rpsL1* Δ*ccnAB* Δ*ccnC* Δ*ccnD* Δ*ccnE* Δ*mntE* | Str^R^ | This study |
| NRD10533 | D39 *rpsL1* Δ*sodA::erm* | Erm^R^ | This study |
| NRD10534 | D39 *rpsL1* Δ*ccnAB* Δ*ccnC* Δ*ccnD* Δ*ccnE* Δ*sodA::erm* | Erm^R^ | This study |
| NRD10769 | TIGR4 *rpsL1* Δ*ccnA* Δ*ccnB* Δ*ccnC* Δ*ccnD* Δ*ccnE* CEP::P_c_-[*kan rpsL^+^*] | Kan^R^ | This study |
| NRD10772 | TIGR4 *rpsL1* Δ*ccnA* Δ*ccnB* Δ*ccnC* Δ*ccnD* Δ*ccnE* CEP::T_1_-T_2_-*ccnA-ccnB* | Str^R^ | This study |
| NRD10787 | TIGR4 *rpsL1* Δ*ccnA* Δ*ccnB* Δ*ccnC* Δ*ccnD* Δ*ccnE* CEP::T_1_-T_2_-*ccnA-ccnB* Δ*bgaA*::*kan-T_1_-T_2_-ccnD* | Kan^R^ | This study |
| TIGR4S | TIGR4 *rpsLK56T* | Str^R^ | This study |
| TIGR4SΔcps | TIGR4 *rpsLK56T* Δ*cps* | Str^R^ | This study |

**Abbreviations**

Kan, kanamycin; Str, streptomycin; Erm, Erythromycin.

**References**

1. Sinha D, Frick JP, Clemons K, Winkler ME, De Lay NR. Pivotal Roles for Ribonucleases in *Streptococcus pneumoniae* Pathogenesis. *mBio*. 2021; 12(5):e0238521. https://doi.org/10.1128/mBio.02385-21 PMID: 34544281

2. Ramos-Montañez S, Tsui HC, Wayne KJ, Morris JL, Peters LE, Zhang F, et al. Polymorphism and regulation of the *spxB* (pyruvate oxidase) virulence factor gene by a CBS-HotDog domain protein (SpxR) in serotype 2 *Streptococcus pneumoniae*. *Molecular Microbiology*. 2008; 67(4):729-46. https://doi.org/10.1111/j.1365-2958.2007.06082.x PMID: 18179423

3. L Sham LT, Jensen KR, Bruce KE, Winkler ME. Involvement of FtsE ATPase and FtsX extracellular loops 1 and 2 in FtsEX-PcsB complex function in cell division of *Streptococcus pneumoniae* D39. *mBio*. 2013; 4(4):e00431-13. https://doi.org/10.1128/mBio.00431-13 PMID: 23860769

4. Sinha D, Zheng JJ, Tsui HT, Richardson JD, De Lay NR, Winkler ME. S1 Domain RNA-Binding Protein CvfD Is a New Posttranscriptional Regulator That Mediates Cold Sensitivity, Phosphate Transport, and Virulence in Streptococcus pneumoniae D39. *Journal of Bacteriology*. 2020; 202(18):e00245-20. https://doi.org/10.1128/JB.00245-20 PMID: 32601068

5. Zafar MA, Kono M, Wang Y, Zangari T, Weiser JN. Infant Mouse Model for the Study of Shedding and Transmission during *Streptococcus pneumoniae* Monoinfection. *Infection and immunity*. 2016; 84(9):2714-22. https://doi.org/10.1128/IAI.00416-16 PMID: 27400721

6. Tettelin H, Nelson KE, Paulsen IT, Eisen JA, Read TD, Peterson S, et al. Complete genome sequence of a virulent isolate of Streptococcus pneumoniae. *Science*. 2001; 293(5529):498-506. https://doi.org/10.1126/science.1061217 PMID: 11463916

7. Bruce KE, Rued BE, Tsui HT, Winkler ME. The Opp (AmiACDEF) Oligopeptide Transporter Mediates Resistance of Serotype 2 *Streptococcus pneumoniae* D39 to Killing by Chemokine CXCL10 and Other Antimicrobial Peptides. *Journal of Bacteriology*. 2018; 200(11):e00745-17. https://doi.org/10.1128/JB.00745-17 PMID: 29581408
