# Supplementary material for: The five homologous CiaR-controlled Ccn sRNAs of Streptococcus pneumoniae modulate Zn-resistance
Source: PLoS Pathog. 2024 Oct 3;20(10):e1012165. doi: 10.1371/journal.ppat.1012165 (PMC11478796; doi:10.1371/journal.ppat.1012165)
Supplement: S2 Table — (DOCX) [file ppat.1012165.s008.docx]

**S2 Table.** Primers used to construct mutants used in this study

| **Primer name** | **Primer Sequence (5'-3')** | **Template** | | **Product** |
| --- | --- | --- | --- | --- |
| **For construction of strain NRD10068 (Δ*ccnA::*P_c_-[kanR-rpsL^+^])** | | | | |
| Spn001 | TCATAGACAAGGCGACTGGTAAGG | IU1945 | | Upstream of *ccnA* |
| Spn002 | CCATTAAAAATCAAACGGATCCTAAAAAAAGTTTAGGATTTTATTAAATAAAGTTAGG |  |  |  |
| kanrpsL For | TAGGATCCGTTTGATTTTTAATGGATAATG | K272 | | P_c_-[*kan rpsL^+^*] |
| kanrpsL rev | GGGCCCCTTTCCTTATGCTTTTG |  |  |  |
| Spn003 | TCCAAAAGCATAAGGAAAGGGGCCCGGCTTTTTGCGTGGTGAGGTGCTGGTG | IU1945 | | Downstream of *ccnA* |
| Spn004 | GGCCCAAATAAGAGCACATGATCC |  |  |  |
| **For construction of strain NRD10069 (Δ*ccnB::*P_c_-[kanR-rpsL^+^])** | | | | |
| Spn005 | ATCATAGACAAGGCGACTGGTAAGGC | IU1945 | | Upstream of *ccnB* |
| Spn006 | CCATTAAAAATCAAACGGATCCTAGGAGGTCTTTATTTAATAACTACATG |  |  |  |
| kanrpsL For | TAGGATCCGTTTGATTTTTAATGGATAATG | K272 | | P_c_-[*kan rpsL^+^*] |
| kanrpsL rev | GGGCCCCTTTCCTTATGCTTTTG |  |  |  |
| Spn007 | TCCAAAAGCATAAGGAAAGGGGCCCCAGGTGGAGTTTTTTAGCTCTATTTCAG | IU1945 | | Downstream of *ccnB* |
| Spn008 | CCAAATGAACACTACGACTACCCTCACC |  |  |  |
| **For construction of strains NRD10070 and NRD10078 (Δ*ccnC::*P_c_-[kanR-rpsL^+^])** | | | | |
| Spn036 | GCCTTATCATATCGAGTTGGATCGCTTGC | IU1945 | | Upstream of *ccnC* |
| Spn034 | CACATTATCCATTAAAAATCAAACGGATCCTAGTCTATAGTATACCCGACCTATCTTAAAC |  |  |  |
| kanrpsL For | TAGGATCCGTTTGATTTTTAATGGATAATG | K272 | | P_c_-[*kan rpsL^+^*] |
| kanrpsL rev | GGGCCCCTTTCCTTATGCTTTTG |  |  |  |
| Spn035 | CGTCCAAAAGCATAAGGAAAGGGGCCCGTGTTGGGATTCATGATATAATAATAAAATCG | IU1945 | | Downstream of *ccnC* |
| Spn037 | TAATCCCCATCAATGACCCCAACTGAGT |  |  |  |
| **For construction of strains NRD10071 and NRD10080 (Δ*ccnD::*P_c_-[kanR-rpsL^+^])** | | | | |
| **Spn020** | AATGAGTTAGAGCCTGGGGATGTCC | IU1945 | | Upstream of *ccnD* |
| **Spn018** | ATCCATTAAAAATCAAACGGATCCTAGAACTTAGTGTACACTCCCTAGCTTAAAGTTTCC |  |  |  |
| kanrpsL For | TAGGATCCGTTTGATTTTTAATGGATAATG | K272 | | P_c_-[*kan rpsL^+^*] |
| kanrpsL rev | GGGCCCCTTTCCTTATGCTTTTG |  |  |  |
| Spn019 | CGTCCAAAAGCATAAGGAAAGGGGCCCGAAAAATGGGCTTGGTGCCTGAGAAT | IU1945 | | Downstream of *ccnD* |
| Spn021 | GTCAGGTAATTCTCCAAGGGAATGG |  |  |  |
| **For construction of strains NRD10072 and NRD10083 (Δ*ccnE::*P_c_-[kanR-rpsL^+^])** | | | | |
| Spn023 | GTATCGGTGACACCTATTTCTCTGA | IU1945 | | Upstream of *ccnE* |
| Spn022 | CACATTATCCATTAAAAATCAAACGGATCCTAGATTATAGTATACACATCTAATCTT |  |  |  |
| kanrpsL For | TAGGATCCGTTTGATTTTTAATGGATAATG | K272 | | P_c_-[*kan rpsL^+^*] |
| kanrpsL rev | GGGCCCCTTTCCTTATGCTTTTG |  |  |  |
| Spn024 | CGTCCAAAAGCATAAGGAAAGGGGCCCTCATTCATGATATAATAGAAGCAAACGGAG | IU1945 | | Downstream of *ccnE* |
| Spn025 | CACTGATTTCAGAACTCTCTACTTCTATCC |  |  |  |
| **For construction of strain NRD10073 (Δ*ccnA*)** | | | | |
| Spn013 | CAGTGGAAAAGCATGCGGAGGATTTG | IU1945 | | Upstream of *ccnA* |
| Spn015 | TCTATCACCAGCACCTCACCACGCGACTATATAATACTAGACCATCCT |  |  |  |
| Spn014 | AGGATGGTCTAGTATTATATAGTCGCGTGGTGAGGTGCTGGTGATAGA | IU1945 | | Downstream of *ccnA* |
| Spn004 | GGCCCAAATAAGAGCACATGATCC |  |  |  |
| **For construction of strain NRD10074 (Δ*ccnB*)** | | | | |
| Spn013 | CAGTGGAAAAGCATGCGGAGGATTTG | IU1945 | | Upstream of *ccnB* |
| Spn028 | GTCCCCAAAAGCCTGAAATAGAGCTAACTACATGATACAAGACGAAACTTAAAAC |  |  |  |
| Spn029 | AAGTTTCGTCTTGTATCATGTAGTTAGCTCTATTTCAGGCTTTTGGGGACTATTC | IU1945 | | Downstream of *ccnB* |
| Spn004 | GGCCCAAATAAGAGCACATGATCC |  |  |  |
| **For construction of strains NRD10075 and NRD10079 (Δ*ccnC*)** | | | | |
| Spn036 | GCCTTATCATATCGAGTTGGATCGCTTGC | IU1945 | | Upstream of *ccnC* |
| 5-ccnCcleanKO Rev | CGATTTTATTATTATATCATGAATCCCAACACGTCTATAGTATACCCGACCTATCTTAAAC |  |  |  |
| 3'ccnCcleanKO For | GTTTAAGATAGGTCGGGTATACTATAGACGTGTTGGGATTCATGATATAATAATAAAATCG | IU1945 | | Downstream of *ccnC* |
| Spn037 | TAATCCCCATCAATGACCCCAACTGAGT |  |  |  |
| **For construction of strains NRD10076 and NRD10081 (Δ*ccnD*)** | | | | |
| Spn020 | AATGAGTTAGAGCCTGGGGATGTCC | IU1945 | | Upstream of *ccnD* |
| Spn031 | TTCTCAGGCACCAAGCCCATTTTTCGAACTTAGTGTACACTCCCTAGCTT |  |  |  |
| Spn030 | CTTTAAGCTAGGGAGTGTACACTAAGTTCGAAAAATGGGCTTGGTGCCTGAGAAT | IU1945 | | Downstream of *ccnD* |
| Spn021 | GTCAGGTAATTCTCCAAGGGAATGG |  |  |  |
| **For construction of strains NRD10077 and NRD10085 (Δ*ccnE*)** | | | | |
| Spn023 | GTATCGGTGACACCTATTTCTCTGA | IU1945 | | Upstream of *ccnE* |
| Spn033 | CCGTCCTCCGTTTGCTTCTATTATATCATGAATGAGATTATAGTATACACATCTAATCTT |  |  |  |
| Spn032 | AAGATTAGATGTGTATACTATAATCTCATTCATGATATAATAGAAGCAAACGGAGGACGG | IU1945 | | Downstream of *ccnE* |
| Spn025 | CACTGATTTCAGAACTCTCTACTTCTATCC |  |  |  |
| **For construction of strain NRD10171 (Δ*ccnAB::*P_c_-[kanR-rpsL^+^])** | | | | |
| Spn001 | TCATAGACAAGGCGACTGGTAAGG | IU1945 | | Upstream of *ccnA* |
| Spn002 | CCATTAAAAATCAAACGGATCCTAAAAAAAGTTTAGGATTTTATTAAATAAAGTTAGG |  |  |  |
| kanrpsL For | TAGGATCCGTTTGATTTTTAATGGATAATG | K272 | | P_c_-[*kan rpsL^+^*] |
| kanrpsL rev | GGGCCCCTTTCCTTATGCTTTTG |  |  |  |
| Spn007 | TCCAAAAGCATAAGGAAAGGGGCCCCAGGTGGAGTTTTTTAGCTCTATTTCAG | IU1945 | | Downstream of *ccnB* |
| Spn008 | CCAAATGAACACTACGACTACCCTCACC |  |  |  |
| **For construction of strain NRD10176 (Δ*ccnAB*)** | | | | |
| Spn001 | TCATAGACAAGGCGACTGGTAAGG | IU1945 | | Upstream of *ccnA* |
| 53-ccnAB For2 | GCAAAATTTAAGGATGGTCTAGTATTATATAGTCAGCTCTATTTCAGGCTTTTGGG |  |  |  |
| 53-ccnAB Rev2 | CCCAAAAGCCTGAAATAGAGCTGACTATATAATACTAGACCATCCTTAAATTTTGC | IU1945 | | Downstream of *ccnB* |
| Spn008 | CCAAATGAACACTACGACTACCCTCACC |  |  |  |
| **For construction of strains NRD10225 and NRD10306 (Δ*ccnA::*P_c_-[kanR-rpsL^+^])** | | | | |
| T4ccnA For | CAGTGGAAAAGCATGCGGAGG | TIGR4 | | Upstream of *ccnA* |
| 5-T4ccnA-rpsL Rev | CATTATCCATTAAAAATCAAACGGATCCTAGACTATATGATACTAGACCATCCTTAAAC |  |  |  |
| kanrpsL For | TAGGATCCGTTTGATTTTTAATGGATAATG | K272 | | P_c_-[*kan rpsL^+^*] |
| kanrpsL rev | GGGCCCCTTTCCTTATGCTTTTG |  |  |  |
| 3-T4ccnA-rpsL For | CAAAAGCATAAGGAAAGGGGCCCGGTACGACGGGCATGTCG | TIGR4 | | Downstream of *ccnA* |
| T4ccnA Rev | GGCTCATGACTTGGACAATGG |  |  |  |
| **For construction of strains NRD10230 and NRD10312 (Δ*ccnA*)** | | | | |
| T4ccnA For | CAGTGGAAAAGCATGCGGAGG | TIGR4 | | Upstream of *ccnA* |
| 5-T4ccnA-cln Rev | CGACATGCCCGTCGTACCGCAGACTATATGATACTAGACCATCCTTAAAC |  |  |  |
| 3-T4ccnA-cln-For | GTTTAAGGATGGTCTAGTATCATATAGTCTGCGGTACGACGGGCATGTCG | TIGR4 | | Downstream of *ccnA* |
| T4ccnA Rev | GGCTCATGACTTGGACAATGG |  |  |  |
| **For construction of strain NRD10247 (Δ*ccnB::*P_c_-[kanR-rpsL^+^])** | | | | |
| T4ccnB For | GCCATTGTCCAAGTCATGAGC | TIGR4 | | Upstream of *ccnB* |
| 5-T4ccnB-rpsL Rev | CATTATCCATTAAAAATCAAACGGATCCTACTACATGATACAAGACGAAACTTAAAACTAGC |  |  |  |
| kanrpsL For | TAGGATCCGTTTGATTTTTAATGGATAATG | K272 | | P_c_-[*kan rpsL^+^*] |
| kanrpsL rev | GGGCCCCTTTCCTTATGCTTTTG |  |  |  |
| 3-T4ccnB-rpsL For | CAAAAGCATAAGGAAAGGGGCCCAGCTCTATTTCAGGATTTTTGGGAC | TIGR4 | | Downstream of *ccnB* |
| T4ccnB Rev | GAGGACATCTCCAGGCTCTAAATC |  |  |  |
| **For construction of strains NRD10249 and NRD10322 (Δ*ccnB*)** | | | | |
| T4ccnB For | GCCATTGTCCAAGTCATGAGC | TIGR4 | | Upstream of *ccnB* |
| 5-T4ccnB-cln Rev | GTCCCAAAAATCCTGAAATAGAGCTAACTACATGATACAAGACGAAACTTAAAACTAGC |  |  |  |
| 3-T4ccnB-cln-For | GCTAGTTTTAAGTTTCGTCTTGTATCATGTAGTTAGCTCTATTTCAGGATTTTTGGGAC | TIGR4 | | Downstream of *ccnB* |
| T4ccnB Rev | GAGGACATCTCCAGGCTCTAAATC |  |  |  |
| **For construction of strains NRD10251 and NRD10330 (Δ*ccnC::*P_c_-[kanR-rpsL^+^])** | | | | |
| T4ccnC For | GCCTTATCATATCGAGTTGGATCGCTTGC | TIGR4 | | Upstream of *ccnC* |
| 5-T4ccnC-rpsL Rev | CATTATCCATTAAAAATCAAACGGATCCTAGTCTATAGTATACCCGACCTATCTTAAAC |  |  |  |
| kanrpsL For | TAGGATCCGTTTGATTTTTAATGGATAATG | K272 | | P_c_-[*kan rpsL^+^*] |
| kanrpsL rev | GGGCCCCTTTCCTTATGCTTTTG |  |  |  |
| 3-cT4cnC-rpsL For | CAAAAGCATAAGGAAAGGGGCCCGTGTTGGGATTCATGATATAATAATAAAATCG | TIGR4 | | Downstream of *ccnC* |
| T4ccnC Rev | CTTCAAGAACAACCTCTTGGTCTG |  |  |  |
| **For construction of strains NRD10254 and NRD10332 (Δ*ccnC*)** | | | | |
| T4ccnC For | GCCTTATCATATCGAGTTGGATCGCTTGC | TIGR4 | | Upstream of *ccnC* |
| 5-T4ccnC-cln Rev | CGATTTTATTATTATATCATGAATCCCAACACGTCTATAGTATACCCGACCTATCTTAAAC |  |  |  |
| 3-T4ccnC-cln-For | GTTTAAGATAGGTCGGGTATACTATAGACGTGTTGGGATTCATGATATAATAATAAAATCG | TIGR4 | | Downstream of *ccnC* |
| T4ccnC Rev | CTTCAAGAACAACCTCTTGGTCTG |  |  |  |
| **For construction of strains NRD10257 and NRD10336 (Δ*ccnD::*P_c_-[kanR-rpsL^+^])** | | | | |
| T4ccnD For | AATGATTTAGAGCCTGGAGATGTCC | TIGR4 | | Upstream of *ccnD* |
| 5-T4ccnD-rpsL Rev | CATTATCCATTAAAAATCAAACGGATCCTAGAACTTAGTGTACACTCCCTAGCTTAAAG |  |  |  |
| kanrpsL For | TAGGATCCGTTTGATTTTTAATGGATAATG | K272 | | P_c_-[*kan rpsL^+^*] |
| kanrpsL rev | GGGCCCCTTTCCTTATGCTTTTG |  |  |  |
| 3-T4ccnD-rpsL For | CAAAAGCATAAGGAAAGGGGCCCGAAAAATGGGCTTGGTGCCTG | TIGR4 | | Downstream of *ccnD* |
| T4ccnD Rev | GTCAGGTAATTCTCCAAGGGAATGG |  |  |  |
| **For construction of strains NRD10261 and NRD10340 (Δ*ccnD*)** | | | | |
| T4ccnD For | AATGATTTAGAGCCTGGAGATGTCC | TIGR4 | | Upstream of *ccnD* |
| 5-T4ccnD-cln Rev | CAGGCACCAAGCCCATTTTTCGAACTTAGTGTACACTCCCTAGCTTAAAG |  |  |  |
| 3-T4ccnD-cln-For | CTTTAAGCTAGGGAGTGTACACTAAGTTCGAAAAATGGGCTTGGTGCCTG | TIGR4 | | Downstream of *ccnD* |
| T4ccnD Rev | GTCAGGTAATTCTCCAAGGGAATGG |  |  |  |
| **For construction of strains NRD10265 and NRD10344 (Δ*ccnE::*P_c_-[kanR-rpsL^+^])** | | | | |
| T4ccnE For | GTATCGGTGACACCTATTTCTCTGA | TIGR4 | | Upstream of *ccnE* |
| 5-T4ccnE-rpsL Rev | CATTATCCATTAAAAATCAAACGGATCCTAGATTATAGTATACACGTCTAATCTTAAATAGAAC |  |  |  |
| kanrpsL For | TAGGATCCGTTTGATTTTTAATGGATAATG | K272 | | P_c_-[*kan rpsL^+^*] |
| kanrpsL rev | GGGCCCCTTTCCTTATGCTTTTG |  |  |  |
| 3-T4ccnE-rpsL For | CAAAAGCATAAGGAAAGGGGCCCTCATTCATGATATAATAGAAGCAAACGG | TIGR4 | | Downstream of *ccnE* |
| T4ccnE Rev | GCTATCTGGTATCATTTCTGGAGCC |  |  |  |
| **For construction of strains NRD10266 and NRD10345 (Δ*ccnE*)** | | | | |
| T4ccnE For | GTATCGGTGACACCTATTTCTCTGA | TIGR4 | | Upstream of *ccnE* |
| 5-T4ccnE-cln Rev | CCGTTTGCTTCTATTATATCATGAATGAGATTATAGTATACACGTCTAATCTTAAATAGAAC |  |  |  |
| 3-T4ccnE-cln-For | GTTCTATTTAAGATTAGACGTGTATACTATAATCTCATTCATGATATAATAGAAGCAAACGG | TIGR4 | | Downstream of *ccnE* |
| T4ccnE Rev | GCTATCTGGTATCATTTCTGGAGCC |  |  |  |
| **For construction of strains NRD10311 and NRD10346 (*rpsL^+^-rpsG^+^-cat*)** | | | | |
| KK265 | GTGAATTGGTCGGAATTGTAGCTAACAG | TIGR4 | | Upstream of *rpsL +* stop codon of *rpsG* |
| KK529 | GCCTCCTAAATTTACCAACGGAAGTGTGCGAATG |  |  |  |
| KK530 | CCGTTGGTAAATTTAGGAGGCATATCAAATGAACT | IU9681 | | *cat* cassette |
| KK531 | CGCATCCTATCTTATAAAAGCCAGTCATTAGGCC |  |  |  |
| KK532 | CTGGCTTTTATAAGATAGGATGCGAAAGCGTTAAG | TIGR4 | | Stop codon of *rpsG* + downstream of *rpsG* |
| KK270 | CAAGGATATCCGTACCAAGGTCGTTAG |  |  |  |
| **For construction of strains NRD10390 and NRD10391 (*CEP::*P_c_-[kanR-rpsL^+^])** | | | | |
| AmiF For | CCAGCTGTGATTGCAAATCTCCATG | IU5122 | | Upstream of *amiF* + downstream of *amiF* |
| TreR Rev | GGCTTCTTGTTCAAATTTTCCCATTTGATTCTC |  |  |  |
| **For construction of strain NRD10393 (*CEP::*T_1_-T_2_-*ccnA-ccnB*)** | | | | |
| 5-AmiF-Ptet for | CTGGCTGACTAGGAGGAAGG | IU5382 | | Upstream of *amiF* + T_1_-T_2_ terminator |
| 5-ccnABCEP_rev | CCAAGTCATCCCTATACAATTATAGGTGGGTAGAAACGCAAAAAGGCCATC |  |  |  |
| M-ccnAB For | GATGGCCTTTTTGCGTTTCTACCCACCTATAATTGTATAGGGATGACTTGG | IU1945 | | 101 nt upstream of *ccnA* + 139 nt downstream of *ccnB* |
| M-ccnAB Rev | GCTCCCTTTTTTAATGGTAACACCGCTGAAGCGACCACAGACC |  |  |  |
| 3-ccnABCEP_ for | AGGTCTGTGGTCGCTTCAGCGGTGTTACCATTAAAAAAGGGAGC | IU5382 | | 26 nt of spd_1082 + downstream of *amiF* |
| 3-TreR-Ptet rev | CCCATTTGATTCTCCTTATACTTGTCAAAGC |  |  |  |
| **For construction of strain NRD10394 (*CEP::*T_1_-T_2_-*ccnC*)** | | | | |
| 5-AmiF-Ptet for | CTGGCTGACTAGGAGGAAGG | IU5382 | | Upstream of *amiF* + T_1_-T_2_ terminator |
| 5-ccnCCEP_rev | CGAACGCCAACCAATTCACTCGTAGAAACGCAAAAAGGCCATC |  |  |  |
| M-ccnC For | GATGGCCTTTTTGCGTTTCTACGAGTGAATTGGTTGGCGTTCG | IU1945 | | 133 nt upstream of *ccnC* + 111 nt downstream of *ccnC* |
| M-ccnC Rev | GCTCCCTTTTTTAATGGTAACACCGCCTCTCTCAAAGCCTCCC |  |  |  |
| 3-ccnCCEP_ for | GGGAGGCTTTGAGAGAGGCGGTGTTACCATTAAAAAAGGGAGC | IU5382 | | 26 nt of spd_1082 + downstream of *amiF* |
| 3-TreR-Ptet rev | CCCATTTGATTCTCCTTATACTTGTCAAAGC |  |  |  |
| **For construction of strains NRD10396 and NRD10397 (Δ*bgaA-kan-*T_1_-T_2_-*ccnD*)** | | | | |
| 5-BgaFor2 | CTGGTGATATCAAAGCAATCCTTGG | IU10508 | | Upstream of bgaA + T_1_-T_2_ terminator |
| 5-ccnDBga Rev | CCATTTCTCCTGCCAATTTTTCTTGGTAGAAACGCAAAAAGGCCATC |  |  |  |
| M-ccnD For | GATGGCCTTTTTGCGTTTCTACCAAGAAAAATTGGCAGGAGAAATGG | IU1945 | | 86 nt upstream of *ccnD* + 55 nt downstream of *ccnD* |
| M-ccnD Rev | GCAACTGGTTTATGAGAAAGTAAGTTCCCCCATTTTCTTCTATCACTAAGC |  |  |  |
| 3-ccnDBga For | GCTTAGTGATAGAAGAAAATGGGGGAACTTACTTTCTCATAAACCAGTTGC | IU10508 | | 1501 nt of *bgaA* + downstream of *bgaA* |
| 3-Bga-rpsLKan Rev | CTGGTTTTTCCTTAGTCAACTGGATACGG |  |  |  |
| **For construction of strains NRD10441 and NRD10443 (Δ*psaR::*P_c_-[kanR-rpsL^+^])** | | | | |
| 5-psaR for | GAGGCTACCCTGCCTCTACTC | | IU1945 | Upstream of *psaR* |
| 5-psaR rpsL rev | CATTATCCATTAAAAATCAAACGGATCCTATAGATAGTCTTCTTTGTTTGGGGTC | |  |  |
| kanrpsL For | TAGGATCCGTTTGATTTTTAATGGATAATG | | K272 | P_c_-[*kan rpsL^+^*] |
| kanrpsL rev | GGGCCCCTTTCCTTATGCTTTTG | |  |  |
| 3-psaR rpsL for | CAAAAGCATAAGGAAAGGGGCCCATTGCAAAACAACTCTATGTCGAG | | IU1945 | Downstream of *psaR* |
| 3-psaR rev | CCAGAGAGCAAGAGCCACTC | |  |  |
| **For construction of strains NRD10442 and NRD10444 (Δ*mntE::*P_c_-[kanR-rpsL^+^])** | | | | |
| 5-mntE for | CCGCATCTTGAAGCATACCAGC | | IU1945 | Upstream of *mntE* |
| 5-mntE rpsL rev | CATTATCCATTAAAAATCAAACGGATCCTACTCAGCTAACTTGAGATTTGAGATAG | |  |  |
| kanrpsL For | TAGGATCCGTTTGATTTTTAATGGATAATG | | K272 | P_c_-[*kan rpsL^+^*] |
| kanrpsL rev | GGGCCCCTTTCCTTATGCTTTTG | |  |  |
| 3-mntE rpsL for | CAAAAGCATAAGGAAAGGGGCCCTGGCAAAATATCTTTCATCAAGAAACC | | IU1945 | Downstream of *mntE* |
| 3-mntE rev | GGAAGAAAGTCATCGAGTTTCAGG | |  |  |
| **For construction of strains NRD10447 and NRD10449 (Δ*psaR*)** | | | | |
| 5-psaR for | GAGGCTACCCTGCCTCTACTC | | IU1945 | Upstream of *psaR* |
| 5-psaR cln rev | CTCGACATAGAGTTGTTTTGCAATTAGATAGTCTTCTTTGTTTGGGGTC | |  |  |
| 3-psaR cln for | GACCCCAAACAAAGAAGACTATCTAATTGCAAAACAACTCTATGTCGAG | | IU1945 | Downstream of *psaR* |
| 3-psaR rev | CCAGAGAGCAAGAGCCACTC | |  |  |
| **For construction of strains NRD10448 and NRD10450 (Δ*mntE*)** | | | | |
| 5-mntE for | CCGCATCTTGAAGCATACCAGC | | IU1945 | Upstream of *mntE* |
| 5-mntE cln rev | GGTTTCTTGATGAAAGATATTTTGCCACTCAGCTAACTTGAGATTTGAGATAG | |  |  |
| 3-mntE cln for | CTATCTCAAATCTCAAGTTAGCTGAGTGGCAAAATATCTTTCATCAAGAAACC | | IU1945 | Downstream of *mntE* |
| 3-mntE rev | GGAAGAAAGTCATCGAGTTTCAGG | |  |  |
| **For construction of strains NRD10533 and NRD10534 (Δ*sodA::erm*)** | | | | |
| 5-sodA For | CAGGGTCAGTTTGAAGTGATGAAGAG | IU1945 | | Upstream of *sodA* |
| 5-sodA-erm Rev | TATTTTATATTTTTGTTCATCTGTAATACCTCTTTTTCTTTCTATATG |  |  |  |
| M-sodA-erm For | GAAAAAGAGGTATTACAGATGAACAAAAATATAAAATATTCTC | NRD10491 | | erm |
| M-sodA-erm Rev | CCTCCAACTATCATTATTTCCTCCCGTTAAATAATAGATAAC |  |  |  |
| 3-sodA-erm For | TAACGGGAGGAAATAATGATAGTTGGAGGGAAGAATTGTTC | IU1945 | | stop codon of *sodA +* downstream |
| 3-sodA Rev | CCATAGTGTTGACGCATGAGTACAG |  |  |  |
| **For construction of strain NRD10769 (*CEP::*P_c_-[kanR-rpsL^+^])** | | | | |
| 5-AmiF-Ptet for | CTGGCTGACTAGGAGGAAGG | | IU11966 | *amiF* |
| KW111 | TATCCATTAAAAATCAAACGGATCCCTCGAGCTTAGCTGACTTCAACCCA | |  |  |
| KW112 | TGGGTTGAAGTCAGCTAAGCTCGAGGGATCCGTTTGATTTTTAATGGATA | | IU5122 | *CEP::*P_c_-[kanR-rpsL^+^ |
| KW113 | CCATATGACCTATAATGAAAAGCGACGGGCCCCTTTCCTTATGCTTTTGG | |  |  |
| KW114 | CCAAAAGCATAAGGAAAGGGGCCCGTCGCTTTTCATTATAGGTCATATGG | | IU11966 | *treR* |
| 3-TreR-Ptet rev | CCCATTTGATTCTCCTTATACTTGTCAAAGC | |  |  |
| **For construction of strains NRD10772 (*CEP::*T_1_-T_2_-*ccnA-ccnB*)** | | | | |
| 5-AmiF-Ptet for | CTGGCTGACTAGGAGGAAGG | | IU11966 | *amiF* |
| 5-CEP Rev | CGAAATTTGTCCTTTCTCGAGC | |  |  |
| M-CEP-ccnAB For2 | ATGTCAATTTTTATGGGAATTAGGCTC | | NRD10396 | *CEP::*T_1_-T_2_-*ccnA-ccnB* |
| M-CEP-ccnAB Rev2 | CGCAAGAAATAAAAGACTGAGAC | |  |  |
| 3-CEP For | TTACCCACTACAAATATTATAGAGCCG | | IU11966 | *treR* |
| 3-TreR-Ptet rev | CCCATTTGATTCTCCTTATACTTGTCAAAGC | |  |  |
| **For construction of strain NRD10787 (Δ*bgaA-kan-*T_1_-T_2_-*ccnD*)** | | | | |
| 5' Bga For | GATATCTGGCACTTGTCTATCACAGGTC | | U11966 | Upstream of bgaA |
| 5-Bga Rev | AGAATACAAGGAAGGCTACTGC | |  |  |
| DM149 | CAACTGGTGATATCAAAGCAATCCTTGG | | NRD10396 | Δ*bgaA-kan-*T_1_-T_2_-*ccnD* |
| M-ccnD Rev | GCAACTGGTTTATGAGAAAGTAAGTTCCCCCATTTTCTTCTATCACTAAGC | |  |  |
| 3-ccnDBga For | GCTTAGTGATAGAAGAAAATGGGGgaacttactttctcataaaccagttgc | | U11966 | downstream of *bgaA* |
| 3-Bga-rpsLKan Rev | CTGGTTTTTCCTTAGTCAACTGGATACGG | |  |  |
| **For construction of strains TIGR4S and TIGR4SΔcps (*rpsLK56T*)** | | | | |
| HE01 | GCCGTAGTCATCTTTCTTGGCATC | IU11966 | | Upstream *rpsL* + 181 nt of *rpsL* with A167C change |
| HE02 | CTGAGTTAGGTTTTGTAGGTGTCATTGTTC |  |  |  |
| HE03 | GAACAATGACACCTACAAAACCTAACTCAG | IU11966 | | 263 nt of *rpsL* with A167C change + downstream |
| HE04 | CTAATTTGAACCCGGGCTAAAGTTAG |  |  |  |
