# Supplementary material for: The five homologous CiaR-controlled Ccn sRNAs of Streptococcus pneumoniae modulate Zn-resistance
Source: PLoS Pathog. 2024 Oct 3;20(10):e1012165. doi: 10.1371/journal.ppat.1012165 (PMC11478796; doi:10.1371/journal.ppat.1012165)
Supplement: S6 Table — (DOCX) [file ppat.1012165.s012.docx]

**S6 Table.** Oligonucleotide primers and probes used for qRT-PCR and northern blots.

| **Primer** | **Primer or probe sequence (5'-3')** | **Gene** |
| --- | --- | --- |
| D39_czcD_For | CGGGCTCTGTTCTAGTCATTT | *czcD* |
| D39_czcD_Rev | CCAGACTCGCTAACAGATTGAT | *czcD* |
| piuB_For | CGGCTCAGTCACAGAAGTTATC | *piuB* |
| piuB_Rev | GCCTAAGAAGAGCCACTCATAC | *piuB* |
| spd_1267_For | TGGATGAGCCTTCATCGAATTTA | *spd_1267* |
| spd_1267_Rev | CACGGTCAACTATGTCCATCAA | *spd_1267* |
| tuf_For | ATCACTGGTGCTGCTCAAA | *tuf* |
| tuf_Rev | CCTGACGTGAAAGAAGGATGT | *tuf* |
| **Probe** |  |  |
| sodA | GCAAGCAAGGCTTCAAGGTCTTCACCGATTTCAGG | *sodA* |
| 5s rRNA | GCGTTCTAGGGCTTAACTTCTGTGTTCGGCATGGG | *rrfA* |
