# Supplementary material for: The five homologous CiaR-controlled Ccn sRNAs of Streptococcus pneumoniae modulate Zn-resistance
Source: PLoS Pathog. 2024 Oct 3;20(10):e1012165. doi: 10.1371/journal.ppat.1012165 (PMC11478796; doi:10.1371/journal.ppat.1012165)
Supplement: Attachment — Submitted filename: Response to reviewers.pdf [file ppat.1012165.s014.pdf]

Response to Editor comments:

The inclusion of the complete set of strains as requested by 2 reviewers in the experiments shown in Figure 1 is essential along with an extended duration of the growth kinetics. An analysis of czcD mRNA expression with or without high levels of zinc would address the question about zinc dependence of regulation. Finally, the question about analysis of bacterial burdens in the blood and tissue of wild type vs mutant systemic infections could highlight important aspects about the role of these sRNAs during in vivo pathogenesis beyond survival curves.

As recommended by the Editor, we have now created and tested the derivative of strain TIGR4 harboring deletions of all five *ccn* genes and complemented with three of them, and we increased the duration of those growth curves (previous Fig. 1, but now Fig. 3). We have now measured expression of *czcD* in the TIGR4 strain containing or lacking the *ccn* genes, which is now included along with the previously presented results obtained from the D39 derived *S. pneumoniae* strains (Fig. 2). We have presented a complete description of our results and analyses of *S. pneumoniae* burden in the blood of mice in the text (L123-125; 145-150). In short, there is no statistically significant difference in bacterial burden in the blood of moribund mice caused by deletion of any of the *ccn* genes. *S. pneumoniae* burden in the blood of moribund mice for *S. pneumoniae* D39 and derived *ccn* mutants is always around 10^{10}, whereas for TIGR4 and derived ccn mutant, it is always ~10^{7}. *S. pneumoniae* burden in the blood of mice is only lower for surviving mice. We did not take samples from any other tissue of mice during infection, and we simply do not have the resources at this time to redo these experiments in order to take additional tissue samples and determine CFUs.

Responses to Reviewers:

Major Issues: Key Experiments Required for Acceptance
Please use this section to detail the key new experiments or modifications of existing experiments that should be absolutely required to validate study conclusions.

Generally, there should be no more than 3 such required experiments or major modifications for a "Major Revision" recommendation. If more than 3 experiments are necessary to validate the study conclusions, then you are encouraged to recommend "Reject".

Reviewer #1: Major points:

1) Fig. 1: It would be useful to employ the complete set of wild-type, deletion and complementation strains in all experiments to exclude potential pleiotropic effects. As in Figure 1A, the complementation with CcnA, B and D is fully sufficient to restore wild-type like growth; this is missing for the TIGR4 serotype (Figs. 1D-F).

We have now constructed the suggested complementation strain, which took about a month to do as this required the creation of new amplicons for generating the insertions or deletions due to the abundance of SNPs between D39 and TIGR4 in the regions of homology and three sequential

transformations. We now show that complementation of TIGR4 with *ccnA*, *ccnB*, and *ccnD* does restore normal growth to the Δ*ccnABCDE* mutant in BHI broth supplemented with Zn (Fig. 3).

2) Introduction and the first section of the results part: the authors should highlight the difference between the two serotypes and the reason for using both strains. Why do you see differences between the Δccn mutant and the wild-type in the TIGR4 serotype and not in the D39 serotype? Here, it would be useful to compare the results with a complemented strain.

We now highlight in the text that we utilized strain D39 in these studies as it is the archetypal *S. pneumoniae* strain (L116-119), having been studied since the 1940's and that we validated our results obtained with strain D39 with TIGR4 as it comes from another major phylogenic lineage and causes a different disease progression in mice (L141-143) as highlighted by the early work of Carlos Orihuela (PMID1462014). Deletion of the *ccn* genes *does* impact the virulence and Zn resistance of strain D39 and TIGR4, but the extent of difference in these phenotypes vary between these two strain backgrounds. The precise cause of the variation in strength of the two phenotypes caused by loss of the *ccn* genes between these strains is not known at the moment and is worthy of future study. I suspect that it is likely due to differences in the gene content between these strains.

Our main reason for using two very different strains in these studies was to determine whether the effect of the Ccn sRNAs on virulence and Zn resistance was isolated to a single strain or a more universal phenomenon in *S. pneumoniae.* Our results point to the latter.

3) Results: please provide a statement on the degree of redundancy among the five Ccn sRNA? Are they different in sequence and structure? And are all of them regulated in the same way by the same two-component system?

We added to the introduction additional information regarding the Ccn sRNAs to the introduction including the degree of similarity in sequence and structure and the degree of activation by the CiaRH two-component system, which varies considerably (L80-87).

4) The time frame of the growth kinetics of S. pneumoniae in various media should be extended (e.g. in Fig 1B). The Δccn strain displays a growth defect, but it remains unclear whether the mutant will reach the wild-type OD. Similarly, in Fig 1C it might be possible that the difference between wild-type and the Δccn mutant more prominent at later growth stages (see also Figs. 6 and 7).

We have extended the growth curves to 10 to 12 h for what was Fig. 1, but is now Fig. 3. The Δ*ccn* mutant does indeed reach a similar OD_{620} as the *ccn*$^{+}$ parental strain in the D39 background, but is slightly reduced in the TIGR4 background.

5) Table 3: The data suggests that the Ccn sRNAs downregulate CzcD expression independently of zinc.

It is possible that the Ccn sRNAs could regulate the *czcD* mRNA, but we have no concrete evidence for that at the moment. I think that the difference in *czcD* expression in BHI broth alone

is indicative of higher level of bioavailable Zn, which is not sufficiently different between strains to be detected by ICP-MS.

Does the abundance of the Ccn sRNAs change after stimulation with high zinc concentrations? This could be tested by northern blotting?

At the moment, we have no consequential evidence that Zn impacts Ccn sRNA expression. I have examined Ccn sRNA expression in *S. pneumoniae* strain D39 with mock treatment vs TPEN addition or 0.4 mM Zn addition for 15 min. TPEN is a divalent cation chelator that has a preference for Zn. For those, experiments addition of Zn had no statistically significant impact on Ccn sRNA expression, whereas TPEN addition led to a statistically significant down-regulation of CcnB (30% reduction, P=0.0301) and CcnE (37% reduction, P=0.266). While TPEN has a preference for Zn, it can also bind other divalent cations.

How can one explain higher intracellular zinc levels in the Δccn mutant, although czcD is highly expressed?

There are limits to CzcD expression. Zn influx can exceed the Zn exporting capacity of CzcD, even when expressed at higher levels. For example, in BHI supplemented with 0.8 mM Zn, *S. pneumoniae* strain D39 is only able to double a few times, before it ceases growth. We are working towards using a Tn-seq based method to identify suppressors of the Zn sensitivity of the Δ*ccnABCDE* mutant strain. Those results might lead to a more satisfactory answer for the reviewer's question.

6) Discussion: it would useful to clarify under which conditions metal intoxication with zinc might be relevant for S. pneumoniae (e.g. phagocytosis by macrophages, combination with oxidative stress). Does this play a role for your pneumonia infection model? This link is missing.

This in an excellent suggestion. We have now incorporated into our discussion the important role that Zn plays during pneumococcal infection of its host. We have further indicated that this could explain why deletion of the *ccn* genes causes reduced *S. pneumoniae* virulence in the murine invasive pneumonia model. See lines 430-449.

Reviewer #2:
- The strains shown in Figure 1 include complementation of ccnCD and ccnABD. Have the authors included complementation strains of ccnE? This would be interesting as deletion of ccnE alone resulted in growth deficiency shown in Fig S1.

We have now included our extensive analysis of the effects of deletions of the *ccn* genes as a supplementary figure (Fig. S2). We have deleted every combination of one, three, or four *ccn* genes from *S. pneumoniae* strain D39 and tested the impact of those on growth inhibition by Zn. The growth of a *S. pneumoniae* strain harboring only *ccnE* is strongly inhibited by Zn. This result convinced us that complementation of the Δ*ccnABCDE* mutant with *ccnE* alone would not significantly improve growth in the presence of excess Zn.

- The authors note that bacterial burden in the blood of some mice infected with the ccn mutant

strain were as low as ~13 CFU/mL. It would be helpful to see any data showing tissue or blood burden for the systemic models of infection. If mice infected with parental and ccn mutant strain bacteria have equal bacterial burden in blood or tissues but are not experiencing symptoms of infection or succumbing to infection, this could be an interesting finding.

We have not examined the CFUs of *S. pneumoniae* strains in any other tissue, except blood. We use CFU number in the blood to validate that mice were euthanized at the appropriate time, i.e., in a moribund state. While CFUs of TIGR4 are always much lower than D39 in this tissue, there remains a strong strain dependent correlation between CFUs and signs of the moribund state of mice. There is no statistically significant difference between the CFUs in the blood of moribund mice between any of the *ccn*$^{+}$ and derived *ccn* mutant strains. We now mention that in the text (L123-125; 145-150). Repeating these experiments to capture CFUs in other tissues is not feasible at this time.

- As written, it is confusing to show data prior to discussion of it in the manuscript. For example, all of Fig 1, S1, and S3 are shown with and without the addition of zinc but not fully discussed until Ln 185-196. This results in a feeling of redundancy when you get to the portion of the results section when this is actually discussed. There is similar confusion in the current placement of the sodA Northern blot that is not mention in the results section at Ln 156-168. The easiest solution for this, I think would be moving Fig 2 and S2 ahead of all growth data. The sodA Northern data should be moved to later in the manuscript where Mn-related genes are mentioned and therefore, relevance of sodA is more apparent.

We have incorporated the criticism of the reviewer by rearranging the order of figures and splitting up the *sodA* data into a later figure. We also condensed the description of our gene expression data for strains grown in BHI supplemented with Zn to ameliorate this impression of redundancy.

- The statement on Ln 222-224 is an important precursor for the result statement on Ln 165-168. Without this introductory prelude, it's unclear how Mn fits into this mechanism.

We presented the results mostly in the chronological order in which they were obtained and presented in the manuscript step-by-step the logic that we used at the time to proceed in out investigations. To address the reviewer's point, I provide more context in of how Mn fits into the picture into the earlier lines (L174-181).

Reviewer #3: none noted.

Minor Issues: Editorial and Data Presentation Modifications
Please use this section for editorial suggestions as well as relatively minor modifications of existing data that would enhance clarity.

Reviewer #1: Minor points:
Line 28: zinc intoxication and mis-metallation is specific to S. pneumoniae? I recommend to re-write the first two sentences more generally.

As suggested, we revised the first two sentences. High levels of Zn can lead to mismetallation of Mn-dependent enzymes in all bacteria, but this is particularly problematic or toxic for Mn-centric bacteria, i.e., bacteria that tend to use Mn instead of Fe as an enzyme co-factor. This includes but is not limited to Firmicutes, e.g., *Mycobacterium tuberculosis* and *Borrelia burgdorferi* are also Mn-centric pathogens.

Line 115: Why in a rpsL1 background strain?

The *rpsL1* and *rpsLK56T* alleles, which are recessive and confer streptomycin resistance, are used for two reasons. Firstly, these alleles facilitate construction of clean deletions using the Janus cassette, which has the selectable kanamycin resistance gene and the counter-selectable wild type *rpsL* allele that confers streptomycin sensitivity. Secondly, the streptomycin resistance conferred by *rpsL1* and *rpsLK56T* allows us to isolate *S. pneumoniae* from mouse tissues after infection. In our hands, the *rpsL1* allele is less resilient in conferring streptomycin resistance in TIGR4 making strain construction at times more difficult, and therefore, we more recently began utilizing a different *rpsL* allele conferring streptomycin resistance, *rpsLK56T* allele, that has been used with TIGR4 (see PMID 32825523) and can provide better streptomycin resistance.

Line 191: “or” instead of “and”
corrected

Line 270-278: redundant

While some of the changes in gene expression between the wild type and *ccn* mutant are the same in BHI broth and BHI broth supplemented with Zn, those results were not presented earlier in the text. We have made other changes in the text to reduce the appearance of redundancy as described in our responses above.

326: swap have and we
The sentence was rewritten.

Line 333: in “the” Zn
Changed

Line 353: strains
changed to plural

Reviewer #2: - Table 2 could be moved to supplement.

We decided to keep Table 2 in the main body of the manuscript as we think that it is important for these results to be more accessible to the reader.

- Figure 3 would benefit from statistical analyses.
Statistical analyses are now provided.

- Ln 389 – edit ‘invasive pneumoniae’ to ‘invasive pneumonia’

The two incidences of this mistake were corrected.

Reviewer #3:

(1) when first describing the complement strategy, spell out the rationale for the strategy of generating Dccn//ccnA+B+D+ and Dccn//ccnC+D+ , and why the followed up with the former. We have provided our rationale for these complementation experiments and now included the data that supports this logic (Figs. S2 and S3). In short, we tested every combination single, triple, and quadruple deletions of the *ccn* genes on *S. pneumoniae* growth in BHI supplemented with 0.2 mM Zn. Strains having just *ccnA* and *ccnB* or *ccnC* and *ccnD* appeared to us to grow the most similar to the ccn^{+} parent strain under these conditions. However, when we attempted to complement of the Δ*ccnABCDE* mutant with *ccnA* and *ccnB* or *ccnC* and *ccnD*, we observed only partial restoration of Zn resistance. Thus, we tested whether complementation of the Δ*ccnABCDE* mutant with *ccnA, ccnB*, and *ccnD* restored growth to that of the ccn^{+} parental strain in the presence of Zn, and it did.

(2) In the description of Figure 5 and Table 2, could the authors address the observation that the complemented strain does not rescue WT phenotype in C+Zn.

Figures S1C and S1D show that the deletion of *ccnE* decreases growth yield compared to the WT parent and likely impairs zinc resistance. However, the genotype for the complemented strain used in the ICP-MS analyses from Figure 5 is D39 *rpsL1* Δ*ccnAB* Δ*ccnC* Δ*ccnD* Δ*ccnE* CEP::T1-T2*ccnA*-*ccnB* Δ*bgaA*::*kan*-T1-T2-*ccnD*. That is, *ccnC* and *ccnE* are not complemented back in this strain. Full restoration of Δ*ccnAB* Δ*ccnC* Δ*ccnD* Δ*ccnE* zinc and manganese levels to the WT levels may require complementation of all the *ccn* genes. This is one possible explanation for the apparent lack of complementation.

We have added a line to results section addressing this finding (L320-322).

(3) Tables with gene expression results (Table S3 & s4) have one column with either name or ID. These are more user friendly, especially for parsing by programs, when one column uniformly has the IDs and another has annotation when available.

We have adjusted Table S3, S4, and S5 to have one column with the locus tag (SPD_# or SP_#), and the gene name if present in the annotation file (.gff).
