# Supplementary material for: The five homologous CiaR-controlled Ccn sRNAs of Streptococcus pneumoniae modulate Zn-resistance
Source: PLoS Pathog. 2024 Oct 3;20(10):e1012165. doi: 10.1371/journal.ppat.1012165 (PMC11478796; doi:10.1371/journal.ppat.1012165)
Supplement: Attachment — Submitted filename: CiaR sRNAs Zn_PlosPath.docx [file ppat.1012165.s013.docx]

The five homologous CiaR-controlled Ccn sRNAs of *Streptococcus pneumoniae* modulate Zn-resistance

NR De Lay, N Verma, D Sinha, A Garrett, MK Osterberg, S Reiling, D Porter, DP Giedroc, and ME Winkler

In this work, De Lay et al., provide evidence that 5 sRNAs from *S. pneumoniae* contribute to systemic infection and are involved in the regulation of zinc stress resistance. They show that mutation of all 5 sRNAs, but not mutation of individual sRNAS, results in increased sensitivity to zinc toxicity, and that this is likely to due to an imbalance of intracellular Zn:Mn that could impact downstream function of superoxide dismutase and result in a sensitivity to oxidative stress. This work provides exciting new information of potential mechanisms of sRNA regulation and the impact on bacterial homeostasis and virulence. This manuscript would benefit from adding more data obtained from the animal studies about bacterial burden – this was very briefly mentioned in the text in the context of the blood, but it would be nice to see tissue data as well. The data presented are interesting, but I found the formatting for the figures and the results to be a bit chaotic and required scanning back-and-forth from different sections - overall, this made the manuscript confusing and led to the feeling of redundancy as you read through the text.

- The strains shown in Figure 1 include complementation of *ccnCD and ccnABD.* Have the authors included complementation strains of *ccnE*? This would be interesting as deletion of *ccnE* alone resulted in growth deficiency shown in Fig S1.
- The authors note that bacterial burden in the blood of some mice infected with the *ccn* mutant strain were as low as ~1^3^ CFU/mL. It would be helpful to see any data showing tissue or blood burden for the systemic models of infection. If mice infected with parental and *ccn* mutant strain bacteria have equal bacterial burden in blood or tissues but are not experiencing symptoms of infection or succumbing to infection, this could be an interesting finding.
- Figure 3 would benefit from statistical analyses.
- As written, it is confusing to show data prior to discussion of it in the manuscript. For example, all of Fig 1, S1, and S3 are shown with and without the addition of zinc but not fully discussed until Ln 185-196. This results in a feeling of redundancy when you get to the portion of the results section when this is actually discussed. There is similar confusion in the current placement of the *sodA* Northern blot that is not mention in the results section at Ln 156-168. The easiest solution for this, I think would be moving Fig 2 and S2 ahead of all growth data. The *sodA* Northern data should be moved to later in the manuscript where Mn-related genes are mentioned and therefore, relevance of *sodA* is more apparent.
- The statement on Ln 222-224 is an important precursor for the result statement on Ln 165-168. Without this introductory prelude, it’s unclear how Mn fits into this mechanism.

Minor:

- Table 2 could be moved to supplement.
- Ln 389 – edit ‘invasive pneumoniae’ to ‘invasive pneumonia’
